# Temporal Changes in the Required Shoe-Floor Friction when Walking following an Induced Slip

**DOI:** 10.1371/journal.pone.0096525

**Published:** 2014-05-02

**Authors:** Danielle N. Beringer, Maury A. Nussbaum, Michael L. Madigan

**Affiliations:** 1 Department of Health and Exercise Science, Wake Forest University, Winston-Salem, North Carolina, United States of America; 2 Department of Industrial and Systems Engineering, School of Biomedical Engineering and Sciences, Virginia Tech, Blacksburg, Virginia, United States of America; 3 Department of Engineering Science and Mechanics, School of Biomedical Engineering and Sciences, Virginia Tech, Blacksburg, Virginia, United States of America; University of Utah, United States of America

## Abstract

Biomechanical aspects of slips and falls have been widely studied to facilitate fall prevention strategies. Prior studies have shown changes in gait after an induced slipping event. As such, most researchers only slip participants one time to avoid such changes that would otherwise reduce the external validity of experimental results. The ability to slip participants more than once, after allowing gait to return to a natural baseline, would improve the experimental efficiency of such studies. Therefore, the goal of this study was to characterize the temporal changes in required shoe-floor friction when walking following an induced slip. Two experiments were completed, and each employed a different potential strategy to promote the return of gait to a natural baseline after slipping. In the first experiment, extended time away from the laboratory was used to promote the return of gait to baseline. We measured required coefficient-of-friction among 36 young adult male participants over four sessions. The first three sessions provided measurements during baseline (i.e., natural gait) both prior to slipping and immediately after slipping. The fourth session provided a measurement 1–12 weeks after slipping. In the second experiment, an extensive number of walking trials was used to promote the return of gait to baseline. We measured required coefficient-of-friction among 10 young adult male participants in a single session. Measurements were collected during 10 baseline walking trials, immediately after slipping, and during 50–55 additional trials. In both experiments, required coefficient-of-friction decreased 12–16% immediately after a single slip, increased toward baseline levels over subsequent weeks/walking trials, but remained statistically different from baseline at the end of the experiments. Based on these results, experiments involving slipping participants multiple times may not have a high level of external validity, and researchers are encouraged to continue to limit experimental protocols to a single induced slip per participant.

## Introduction

Falls are a significant source of unintentional injuries and medical costs in the United States. In 2005, more than 8.7 million emergency department visits were made to U.S. hospitals due to fall-related injuries, making up 20% of all injury visits [1]. Additionally, the National Safety Council reported that falls are the second-leading cause of unintentional death in homes and communities, resulting in more than 25,000 fatalities in 2009 [2]. Claims for fall-related occupational injuries constitute about 25% of all workers' compensation costs in the U.S., which is estimated to total more than $6 billion annually [3]. The large number of fall-related injuries and high associated medical costs highlight the importance of research into the mechanisms and prevention of falls [4].

Slipping is responsible for a large proportion of falls [3], [5]–[7]. For example, Courtney et al. (2001) reported that slipping contributed to 40-50% of reported fall-related injuries [3]. The causes of slips are complex, involving the interaction of numerous factors, both intrinsic (human factors) and extrinsic (environmental factors) [7]–[10]. The frictional properties of the interface between the shoe and floor are the primary environmental determinants of a slipping event [8], [10]. In particular, the available coefficient of friction between the shoe and floor is determined largely by shoe and floor materials and environmental conditions. The required coefficient of friction (RCOF) is the minimum coefficient of friction necessary at the shoe-floor interface to prevent slipping [11], and is determined from the ratio of shear to normal ground reaction forces during stance. The local maximum of this ratio at 10-20% of stance is typically used as the RCOF, because it is at this point that slipping is thought to most likely to lead to a fall [11]. The risk of slipping increases when the RCOF approaches or exceeds the available COF [7], [8], [11]. However, most researchers only determine the RCOF when assessing the risk of slipping, since obtaining reliable measurements of the available COF between the shoe and floor is challenging [11]. Increases in RCOF indicate an increased risk of slipping.

The biomechanics of slips are commonly studied in laboratory settings in an effort to improve the understanding of slip mechanisms, slip and fall prevention strategies, and risk assessment methods [5], [10]. For example, the RCOF has been correlated with several kinematic gait parameters [5], [11]–[17]. One challenge in studying slips is to maintain natural gait patterns during testing in the laboratory, yet multiple studies have demonstrated that gait changes after slipping [5], [10], [12]. Such changes suggest that any experimental results obtained after gait has been altered due to a prior slip may have limited external validity with respect to natural gait. Therefore, most researchers only slip participants once when it is important to maintain external validity with natural walking [5], [10], [12], [18]–[20]. Describing the temporal changes in gait after an induced slip may allow participants to be slipped more than once, but after an appropriate delay to allow gait to return to a natural baseline. This could substantially improve the efficiency of such experiments, as fewer participants would be needed, thereby reducing the time and resources for participant recruitment, medical screenings, and experimentation. It would also allow the use of within-subject experimental designs (or at least repeated measures for a subset of factors), which have improved statistical power over between-subject designs [21].

Therefore, the goal of this study was to characterize the temporal changes in RCOF when walking following an induced slip. RCOF was used to quantify changes in gait due to its association with risk of slipping [7], [8], [11]. Results from this study could aid in the experimental design of future studies involving laboratory slips, and could allow researchers to slip participants more than once, while ensuring results are descriptive of natural, unexpected slips.

## Materials and Methods

Two separate experiments were completed, with a separate sample in each. All participants were young male adults recruited from the university population, and were free from self-reported musculoskeletal and neurological disorders that may affect gait or balance.

### Ethics Statement

Prior to any data collection, all participants provided written informed consent by reviewing and signing a consent form that described the aims and procedures of the study. The study procedures, including the consent form, were approved by the Virginia Tech Institutional Review Board.

### Experiment 1

The goal of Experiment 1 was to characterize the temporal changes in RCOF over several weeks after an induced slip. Thirty-six young adult males (mean age = 20.7±2.3 years; height  = 1.80±0.08 m; mass = 79.8±11.8 kg) were recruited from the local university population. Participants first completed three baseline sessions on separate days to determine baseline RCOF during normal walking. During the third session, and after RCOF measurements, participants were exposed to an unexpected slip. Additional walking trials were then repeated for approximately 15 minutes to assess changes in RCOF immediately after slipping. Participants then returned for a fourth session either one, two, four, six, or twelve weeks after slipping to measure RCOF. Participants were recruited in groups of three to five young adult males, and all members of each of these groups returned for the fourth session after the same number of weeks. The first group of participants returned for the fourth session one week after slipping, and subsequent groups returned after progressively more weeks. Participants in the group that returned four weeks after slipping were statistically significantly older compared to the other groups (likely because the oldest participant, aged 29, was in the group), but this group exhibited no other differences in height, mass, or baseline RCOF compared to the other groups.

At the start of the first session, participants were made aware of the possibility of an induced slip during any walking trial or session throughout the experiment. Participants donned laboratory-provided, soft-soled walking shoes to prevent variation in the frictional properties of the shoe-floor interface between participants, and wore a safety harness attached to a track above the walkway to prevent a fall. To start the experiment, participants were asked to walk at a purposeful speed (slightly faster than comfortable) along a 9 meter walkway covered in vinyl flooring. We chose this purposeful speed, rather than a slower comfortable speed, based upon our observations during pilot work that some participants walked at a slower than normal speed once they were informed that a slip may occur. Walking more slowly decreases RCOF [22] and can make it less likely that participants slip when exposed to a slippery floor. Participants were required to maintain a gait speed between 1.5 and 2 m/s during all walking trials, and trials not within a fixed speed range (−0.0525 m/s to +0.0975 m/s from the participant's mean speed) were repeated, with verbal feedback from the investigators to increase or decrease speed. Gait speed was experimentally controlled to avoid changes in speed after slipping from confounding the measurements of RCOF. Participants were given three practice trials at the beginning of each session to adjust to the environment and re-establish their gait speed from the first session (if necessary). After the self-selected purposeful gait speed was determined during the first session, data from approximately 10 acceptable walking trials (appropriate speed and foot placement with respect to a force platform) were collected. During these and all other walking trials, participants were attempting to retain a memorized set of letters, numbers, or symbols presented on note cards before each trial, to divert their attention from walking and a potential slip. Once reaching the far end of the walkway, participants were instructed to sit on a stool with their back to the walkway and memorize a new set of information until notified to turn around (approximately 1.5 minutes) and prepare for the next trial. Session two and the beginning of session three each involved data collection during approximately 10 more acceptable walking trials at the appropriate speed.

After the initial gait trials during session three, a thin layer of vegetable oil was applied with a paint roller to a middle portion of the walkway while the participants had their back to the walkway and were distracted with the memorization task. To minimize auditory or visual cues of the contaminant, participants wore noise protection earmuffs, nature sounds were played, and the lighting was dimmed throughout all sessions. Slips of the stance foot of at least 3 cm during early stance were characterized as a successful slip. If participants were unsuccessfully slipped, the walkway and shoes were cleaned and dried, and another slip was attempted after a few additional walking trials. After a successful slip trial, the walkway and shoes were cleaned and dried, restoring their original state, and 10 additional walking trials were performed. All participants who failed to slip during the first attempt were successfully slipped several trials later in a second slip attempt. A successful slip on the first attempt, or second attempt after a failed first attempt, were assumed to have the same effect on gait because in each case the participant experienced a successful slip. The fourth session was one, two, four, six, or twelve weeks after slipping, and involved approximately 10 additional walking trials.

### Experiment 2

The goal of Experiment 2 was to characterize the temporal changes in RCOF over a number of walking trials performed immediately after slipping during the same session as slipping. Ten young adult males (mean age  = 21.8±1.8 years; height  = 1.81±0.07 m; mass  = 76.1±7.4 kg) completed this experiment. Unlike Experiment 1, these participants completed only one experimental session. Approximately ten trials were performed to determine baseline RCOF during normal walking. Participants were then exposed to an unexpected slip, followed by 50–55 additional walking trials after removing the contaminant from the floor/shoes.

At the start of the session, participants were made aware of a possible slip during any walking trial throughout the experiment. Participants donned laboratory-provided, soft-soled walking shoes to prevent variation in the frictional properties of the shoe-floor interface between participants, and wore a safety harness attached to a track above the walkway to prevent a fall. As in Experiment 1, the experiment started by asking participants to walk at a purposeful speed (slightly faster than comfortable) along a 9 meter walkway covered in vinyl flooring. The same methods were used here as in Experiment 1 to achieve and maintain a gait speed between 1.5 and 2 m/s during all walking trials. After the self-selected purposeful gait speed was determined, data from approximately 10 acceptable walking trials (appropriate speed and foot placement with respect to a force platform) were collected. As in Experiment 1, participants attempted to retain a memorized set of letters, numbers, or symbols during all trials to divert their attention from walking and a potential slip, and earmuffs were worn with background sounds and dimmed lighting. After 10 acceptable walking trials, participants were slipped as described as in Experiment 1. The walkway and shoes were then cleaned and dried, and 50–55 additional walking trials were performed. The number of post-slip trials was selected so that the entire session did not last longer than two hours.

### Analysis

During each trial, the three-dimensional positions of selected anatomical landmarks were sampled at 100 Hz using a six-camera Vicon motion analysis system (Vicon Motion Systems Inc., Centennial, CO), and ground reaction forces were sampled at 1000 Hz using a force platform (Bertec Corporation, Columbus, OH). Markers were placed over the inferior tip of the right scapula, the heel and tip of each shoe, and the lateral malleolus and lateral femoral epicondyle of each lower extremity. Marker position and force platform data were low-pass filtered at 5 and 7 Hz, respectively, using an eighth-order zero-phase-shift Butterworth filter [16]. The RCOF was the primary dependent variable because it is believed to best reflect aspects of gait that contribute to the potential for slipping [5]. It was calculated from the filtered force platform data as a local maximum of the ratio of shear vs. normal ground reaction forces observed during 10–20% of the stance phase of gait [11]. Large values of RCOF that occurred at the beginning and end of stance phase, due to small values of vertical GRF, were considered spurious and ignored. Gait speed and step length were also determined. Gait speed was determined as the mean forward speed of the marker on the right scapula, and step length was determined as the distance between heel contact and contralateral-limb heel contact.

For Experiment 1, a three-way mixed-model ANOVA (independent variables were trial and session as fixed effects, and subject as a random effect) with planned contrasts was used to investigate differences in the dependent variables between sessions. Planned contrasts compared the dependent variables between the three baseline sessions (all three considered together) and each post-slip session. For Experiment 2, a two-way repeated-measures ANOVA (independent variables were trial as a fixed effect, and subject as a random effect) with planned contrasts was used to investigate differences in the dependent variables between trials. Planned contrasts compared the dependent variables between the 10 baseline trials (all 10 considered together) and groupings of five consecutive post-slip trials (first five trials after slipping, second five trials after slipping, etc.). Statistical analyses were performed using JMP 9 (SAS Institute Inc., Cary, NC) with a significance level of *p* = 0.05, and summary values are reported as least squares means ± standard error.

## Results

### Experiment 1

**Figure 1:**
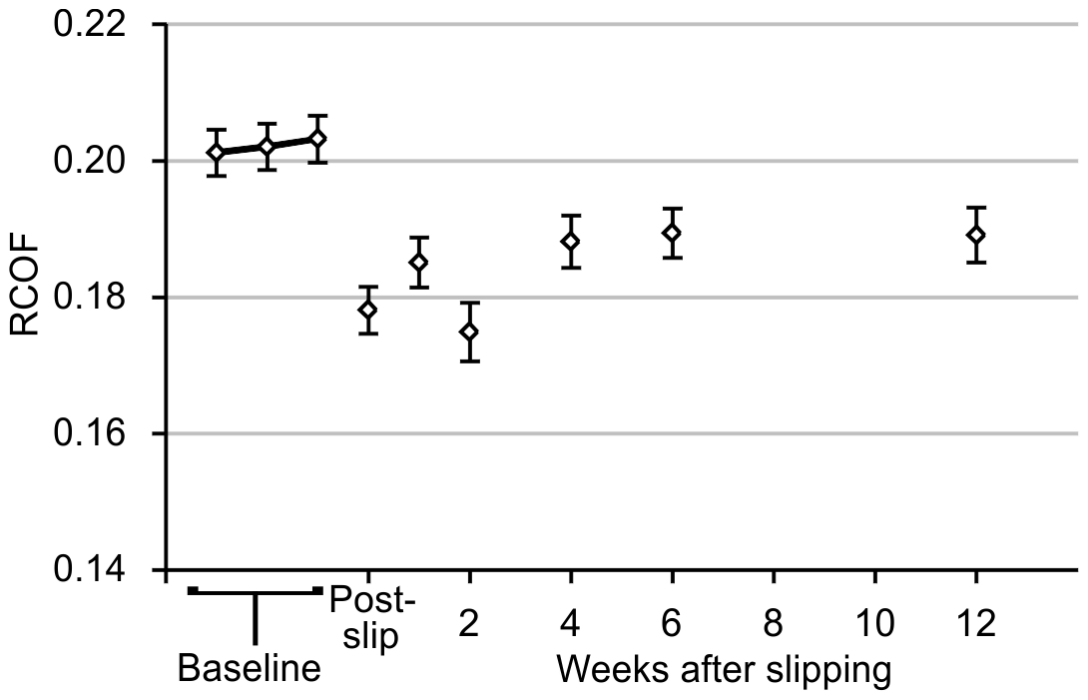
Required coefficient-of-friction across all participants of Experiment 1. Least-square means are shown, and error bars indicate standard error. RCOF was significantly different from baseline at all times after slipping. Post-slip indicates RCOF values immediately after slipping (during the third baseline session).

**Figure 2:**
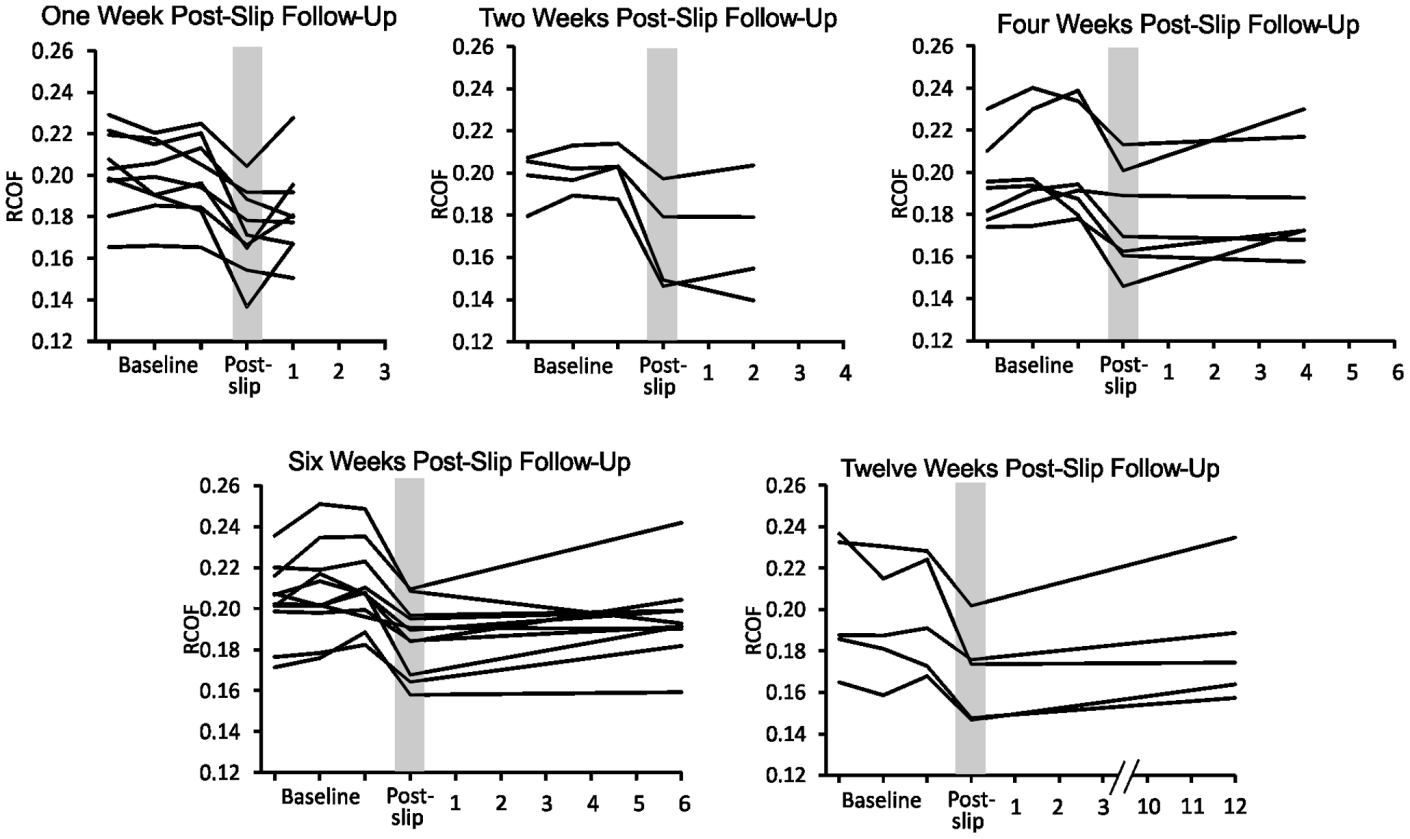
RCOF trends for each participant in Experiment 1. These plots illustrate the variability of RCOF values and temporal changes between participants. As the time after slipping increased, the percentage of participants who exhibited an increase back toward baseline increased. Post-slip indicates RCOF values immediately after slipping (during the third baseline session).

Prior to slipping, the mean RCOF across the three baseline sessions was 0.202±0.003 (Figure 1). Immediately after slipping, RCOF decreased 12% to 0.178±0.003 (*p*<0.001), and exhibited a general increasing trend back toward baseline over the subsequent 12 weeks. However, all post-slip RCOF values remained statistically different from baseline (*p*<0.001). To better illustrate the varying trends in RCOF between participants over all sessions, data from each individual participant are shown in Figure 2. All but one of the 36 participants demonstrated a decrease in RCOF immediately after slipping, and the percentage of participants who showed an increase in RCOF toward baseline increased as the number of weeks between slipping and the fourth session increased. One week after slipping, 44% (four out of nine) of participants showed an increase in RCOF toward their baseline value. Two weeks after slipping, 50% (two out of four) of participants showed an increase in RCOF toward their baseline value. Four weeks after slipping, 57% (four out of seven) of participants showed an increase in RCOF toward their baseline value. Six weeks after slipping, 73% (eight out of eleven) of participants showed an increase in RCOF toward their baseline value. Twelve weeks after slipping, 100% (five out of five) participants showed an increase in RCOF toward their baseline value.

Walking speed and step length did not exhibit any systematic trends over all sessions. Walking speed was 1.56±0.004 m/s during baseline sessions, was 0.4% faster than baseline immediately after slipping (*p* = 0.014), not different (*p* = 0.259) from baseline one week after slipping, 1.6% slower than baseline two weeks after slipping (*p*<0.001), and not different from baseline for the remaining sessions (*p* = 0.153 at four weeks, *p* = 0.544 at six weeks, and *p* = 0.109 at 12 weeks). Step length was 0.82±0.003 m during baseline sessions, was not different from baseline immediately after slipping (*p* = 0.056) or one week after slipping (*p* = 0.224), 2.2% shorter than baseline two weeks after slipping (*p*<0.001), and not different from baseline for the remaining sessions (*p* = 0.397 at four weeks, *p* = 0.112 at six weeks, and *p* = 0.622 at twelve weeks).

### Experiment 2

**Figure 3:**
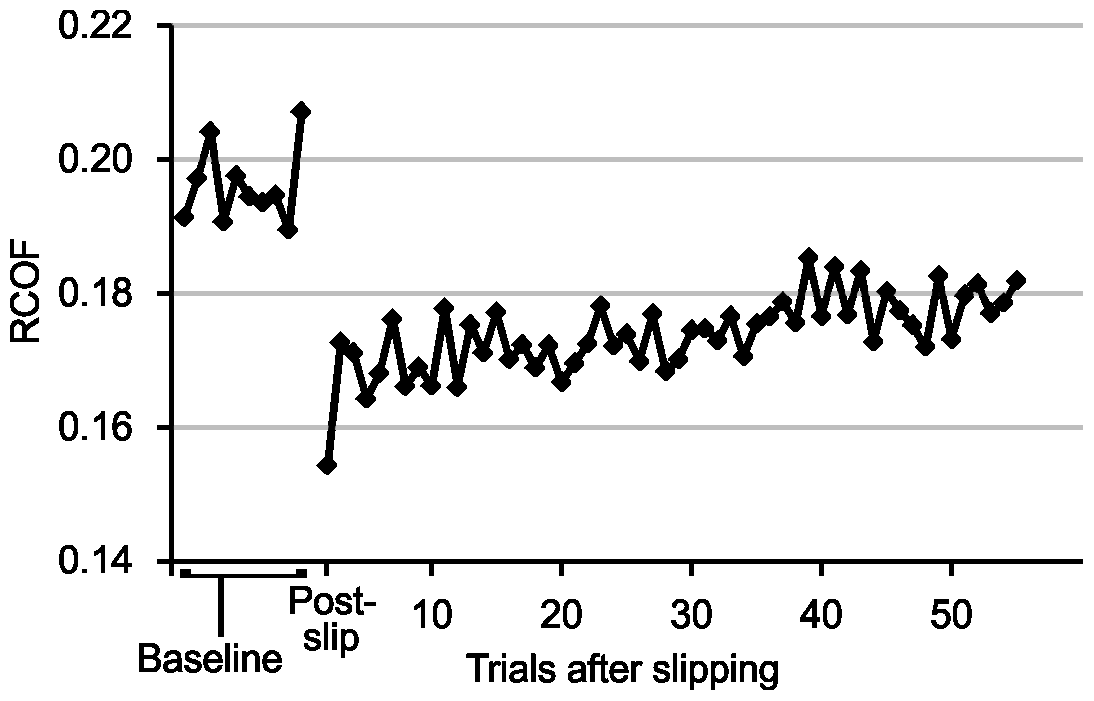
RCOF (least square means) across all participants in Experiment 2. Error bars not included for clarity. RCOF was significantly different from baseline for all trials after slipping. Post-slip indicates RCOF values immediately after slipping.

**Figure 4:**
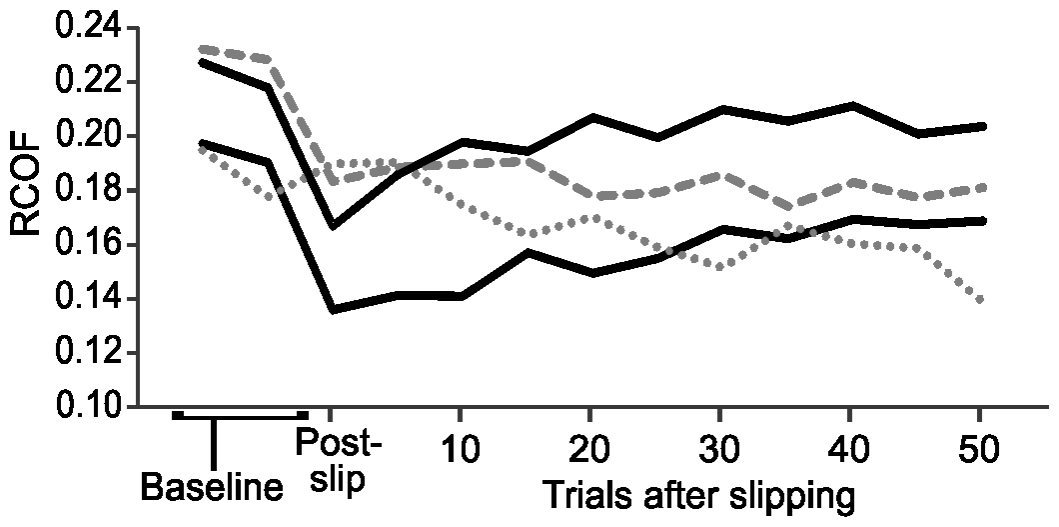
RCOF (least square means) for four representative participants in Experiment 2. Each data point is a mean across five consecutive trials (trials 1–5 after slipping, trials 6–10 after slipping, etc.). The two participants whose RCOF values did not trend back toward baseline after slipping are shown by dotted and dashed lines. Post-slip indicates RCOF values immediately after slipping.

Prior to slipping, the mean RCOF across the 10 baseline trials was 0.196±0.007 (Figure 3). Over the first five trials after slipping, RCOF decreased 16% to 0.166±0.008 (*p*<0.001), and exhibited a general increasing trend back toward baseline over the 55 trials after slipping. However, all post-slip RCOF values remained statistically different from baseline (*p*<0.001). To better illustrate the varying trends in RCOF between participants over all trials, RCOF for four representative participants is shown in Figure 4. Nine of ten participants demonstrated a decrease in RCOF immediately after slipping, and a linear regression fit to each participant's 50–55 trials after slipping showed a positive slope for eight of 10 participants. The two participants whose RCOF did not increase back toward baseline are shown in Figure 4. The predicted RCOF value from the eight linear regression equations with a positive slope after 55 post-slip trials averaged 95.5% of respective baseline RCOF value.

Walking speed and step length did not exhibit the same general trend as RCOF. Walking speed was 1.51±0.035 m/s during baseline sessions, was up to 4.0% faster than baseline (up to 1.57 m/s) over the first 25 trials after slipping (*p* = 0.001–0.033), and not different from baseline over the remaining 25–30 trials (*p* = 0.192–0.949). Step length was 0.81±0.02 m during baseline sessions, did not differ from baseline during the first five trials after slipping, and sporadically exhibited differences from baseline over the 50–55 trials after slipping (*p* = 0.278–0.008) with no systematic changes toward or away from baseline.

## Discussion

The goal of this study was to characterize the temporal changes in RCOF when walking following an induced slip. In Experiment 1, RCOF decreased by a mean of 12% immediately after slipping, and gradually increased toward a pre-slip baseline over the next 12 weeks. However, the RCOF remained statistically lower than baseline for all follow-up sessions up to 12 weeks after slipping. In Experiment 2, RCOF decreased by a mean of 16% immediately after slipping, and gradually increased toward baseline over the next 50–55 trials. However, the RCOF remained statistically lower than baseline for all of these trials. These results indicate that: 1) waiting 12 weeks after slipping, for a potential follow-up experimental session to retest participants, is not sufficient for gait to return to baseline; and 2) repeating 50–55 walking trials after slipping during the same experimental session is not sufficient for gait to return to baseline.

The observed 12–16% decrease in RCOF immediately after slipping is consistent with prior studies and suggests the use of a more cautious gait to reduce the risk of slipping. Cham and Redfern (2002) reported a 5–12% decrease in RCOF from baseline (mean baseline RCOF = 0.18) after slipping while walking over level ground, even though participants were assured they would not be slipped again [5]. Lockhart et al. [13] and Siegmund et al. [23] reported a 20% and 7% decrease in RCOF, respectively, after exposure to a slip. Changes in gait speed and step length immediately after slipping have not been consistently reported. Cham and Redfern (2002) reported no change in stride length after slipping, and gait speed was neither controlled nor reported [5]. Lockhart et al. (2007) reported an 18% decrease in step length, asked participants to walk at their self-selected speed during all trials, and did not report results with respect to gait speed. Heiden et al (2006) reported no change in step length or gait speed after experiencing a slip [12]. We chose to control gait speed to within a small range of each participant's self-selected speed because participants slowed their gait after slipping during pilot testing, and we did not want changes in gait speed to confound our results with respect to RCOF. Controlling speed, however, may have mitigated changes in step length compared to other studies.

Across both Experiments, RCOF decreased an average of 0.028, or 14.1%, immediately after slipping. A change in RCOF of this magnitude can have a substantial effect on the probability of slipping, depending on how close RCOF and the available COF (ACOF) are in magnitude. Burnfield and Powers (2006) demonstrated that it is possible to predict a slip event based on the difference between the ACOF and RCOF [24]. They reported a 5% probability of a slip occurring when the RCOF was 0.047 lower than the ACOF, and a 50% probability of a slip occurring when the RCOF was 0.006 greater than ACOF [24]. These authors also note that, depending upon ACOF, an increase in RCOF as small as 0.05 can contribute to a substantial increase in risk of slipping. Therefore, the mean decrease in RCOF of 0.028 observed in this study would seem to be practically relevant in terms of the risk of slipping.

Experiments 1 and 2 were designed to characterize not only the temporal changes in RCOF after slipping, but also to serve as sample experimental designs that could be employed in future studies in which participants are exposed to more than one slip while allowing for a sufficient “wash out” period for gait to return to a natural baseline. With this in mind, both experimental designs aimed to maximize experimental efficiency by minimizing the number of sessions required from each participant. Experiment 1 aimed to allow RCOF to return to baseline during time away from the lab, and in application would only require one experimental session for each slip (with the necessary time for RCOF to return to baseline between consecutive sessions). Experiment 2 aimed to allow RCOF to return to baseline through repeated trials during a single experimental session, and in application would only require one experimental session for multiple slips (with the necessary number of trials for RCOF to return to baseline between slips). An alternative experimental design we considered, but elected not to pursue, would have required participants to complete multiple post-slip sessions at shorter intervals (1–2 days apart) until RCOF returned to baseline. This design may have resulted in RCOF returning to baseline more quickly than we found in Experiment 1, but would be inefficient in that it would likely require multiple experimental sessions for RCOF to return to baseline.

The RCOF of some participants returned to near baseline, while others did not (Figures 2 and 4). Given this inter-participant variability, it may be possible to track each participant's temporal changes in RCOF, and perform repeated slips on only those whose RCOF returned to baseline. However, we did not pursue this approach because we felt it to be experimentally inefficient since the testing on many participants whose RCOF does not return to near baseline would contribute to wasted time and resources. Future studies could better evaluate the costs and benefits of this approach.

The results from Experiment 1 showed that post-slip gait adaptations indicative of a lower risk of slipping persisted for at least 12 weeks (although the magnitude of these adaptations waned with time). While this time duration can be viewed as a challenge for researchers desiring to slip participants multiple times, it provides support for training interventions for improved slip prevention. Researchers have proposed that slipping or tripping individuals periodically in a controlled environment is a training intervention that can alter gait to reduce the risk of slips and trips, or improve balance recovery ability [25]–[27]. Our results indicate that changes in gait induced by a single slip last up to 12 weeks, which suggests that a slip or trip training intervention may also have lasting benefits.

Several limitations of this study warrant discussion. First, it is unclear if the slip-induced changes in RCOF observed in a laboratory setting generalize to outside of the laboratory. The informed consent process used in this study made participants aware of a potential slip, which may have a heightened their awareness of slipping more than is typical in a natural environment. As such, it is possible that the changes in RCOF were limited to the laboratory. Second, this study only investigated changes in gait after slipping. Therefore, no conclusions can be made about the temporal changes in balance recovery capability following a slip. Third, this study was limited to young adult males to avoid potential age and gender effects. It is unclear if the temporal changes in RCOF would differ among other populations. Fourth, we investigated the temporal changes in RCOF after a single slip. It is unclear if the temporal changes in RCOF after an additional slip would differ from after the initial slip.

In conclusion, RCOF during gait decreased 12–16% immediately after a single slip, tended to return toward baseline over subsequent weeks/walking trials, but remained statistically different from baseline at the end of our experiments. Given these results, experiments involving slipping participants multiple times do not appear practical at this time, and slip researchers are encouraged to continue to limit slips to one per participant to maintain high external validity of their results. Although other experimental designs involving more sessions per participant may help to induce RCOF to return to baseline more quickly, these would likely be less efficient and thus take away from the benefits of slipping research participants more than once. Our results also provide support for the use of perturbation-based balance training for reducing fall risk, as the changes in RCOF, which suggest a lower risk of slipping, persisted for an extended period of time.
